# When an enzyme isn’t just an enzyme anymore

**DOI:** 10.1093/jxb/erx080

**Published:** 2017-04-28

**Authors:** Brenda S.J. Winkel

**Affiliations:** 1 Department of Biological Sciences, Virginia Tech, Blacksburg, VA 24060, USA

**Keywords:** Arogenate dehydratase, chloroplast division, moonlighting proteins, nuclear localization, phenylalanine biosynthesis, stromules

## Abstract

This article comments on:

Bross CD, Howes TR, Abolhassani Rad S, Kljakic O, Kohalmi SE. 2017. Subcellular localizations of Arabidopsis arogenate dehydratases suggest novel and non-enzymatic roles. Journal of Experimental Botany 68, 1425–1440.

**In this issue (pages 1425–1440) Bross *et al.* provide evidence of surprising alternative functions for two isoforms of the plastid enzyme arogenate dehydratase. This study points to a previously unsuspected connection between central metabolism and chloroplast division and a potential new mechanism for retrograde signaling. These findings add to a growing awareness of the complexities of protein function that has substantial implications for both basic and applied plant science.**

We are in the midst of a paradigm shift in our understanding of cellular metabolism, as growing numbers of proteins with well-established catalytic roles are being found to have additional, alternative functions. Although the pervasiveness of this so-called ‘moonlighting’ phenomenon is not yet clear, the MoonProt database (www.moonlightingproteins.org) already has almost 300 entries for multifunctional proteins from across the phylogenetic spectrum, including many originally identified as enzymes of central metabolism. The rapid pace with which new and alternative functions are being reported suggests that current knowledge represents the tip of an iceberg.

## Linking biological processes

An emerging theme surrounding moonlighting proteins is that multi-functionality can provide a powerful mechanism to link diverse cellular processes, including across cellular compartments. Glyceraldehyde-3-phosphate dehydrogenase (GAPDH), a cytoplasmic glycolytic enzyme, is a remarkable example. GAPDH protein has been found to be localized to a wide range of sites in many different organisms, from microbes to plants and animals (He *et al.*, 2013; Zaffagnini *et al.*, 2013; Sirover, 2014). At these various sites GAPDH has been found to participate not only in glycolysis, but in pathogenesis, receptor-mediated cell signaling, iron metabolism, histone biosynthesis, and control of cell cycle progression (Frederikse *et al.*, 2016), while also serving as a redox sensor and transducer. In these ways, GAPDH is able to integrate multiple biological processes in response to changes in the metabolic and redox status of the cell, a role with an apparently ancient evolutionary origin (Hildebrandt *et al.*, 2015).

There are numerous other examples of glycolytic enzymes with alternative non-catalytic functions, though none as intensively studied as GAPDH (Jeffery, 2014). The functions of these proteins range from serving as transcriptional regulators that are sensitive to sucrose concentrations to binding of plasminogen during microbial pathogenesis (Rolland *et al.*, 2006; He *et al.*, 2013; Henderson and Martin, 2013; Vega *et al.*, 2016). Dual functionality is also associated with enzymes of many other pathways of central metabolism, such as homocitrate synthase, which is essential for lysine biosynthesis in mitochondria but can also mediate DNA repair in the nucleus (Torres-Machorro *et al.*, 2015). Aconitase is another well-established example, functioning not only as a key enzyme in the TCA cycle, but also as an iron-responsive protein that enhances synthesis of proteins involved in iron uptake under low intracellular iron concentrations, a role conserved from microbes to plants and animals (Commichau and Stulke, 2008; Marondedze *et al.*, 2016). Multifunctional proteins are also behind the increasingly apparent connection between central metabolism and chromatin dynamics (Keating and El-Osta, 2015). For example, the mammalian NCOAT enzyme that removes GlcN modifications from nuclear and cytoplasmic proteins also functions as a histone acetyltransferase affecting chromatin status (Toleman *et al.*, 2004). Similarly, a recent screen for proteins that function in heterochromatin regulation revealed a novel role for glutamate dehydrogenase in telomeric silencing (Su and Pillus, 2016).

## The special case of the plant cell

The unique architecture of the plant cell, which includes plastids as well as other structures present only in plants, together with an unusually complex metabolism, suggests that plant evolution has offered unique opportunities for recruitment of enzymes into novel functions. Indeed, plants are proving to be invaluable platforms for the discovery and characterization of multifunctional proteins, often pointing to novel accessory activities not present in other organisms. For example, in Arabidopsis, the L10 ribosomal protein was found to function not only in protein synthesis in the cytosol, but also to contribute to the restriction of virus infection in the nucleus in an as-yet-undetermined manner; this is distinct from the moonlighting functions identified for L10 and other ribosomal proteins in microbes, yeast, Drosophila, and humans, although in both plants and humans phosphorylation may be involved in directing L10 to the nucleus (Warner and McIntosh, 2009).

Plants have also provided intriguing examples of closely related genes encoding proteins with a shared catalytic function but a diversity of accessory functions, as in the case of the germin and germin-like proteins (Li *et al.*, 2016). These superoxide dismutases exhibit a wide array of alternative functions, including as oxalate oxidases, as auxin receptors, and as polyphenol oxidase and serine protease inhibitors in a variety of plant species. In another extension of work in other organisms, studies in Arabidopsis link inositol metabolism to plant defense through histone remodeling (Latrasse *et al.*, 2013). In this case, myo-inositol phosphate synthase, a cytoplasmic enzyme, interacts with the histone methyltransferases ATXR5 and ATXR6, in the nucleus, controlling its own expression via histone modification, including in response to pathogens. Reports of novel localization of components of well-established pathways continue to mount in plants, suggesting that there are many more dual functionalities waiting to be uncovered, including among the enzymes of specialized metabolism (Box 1).

**Box 1. The potential for moonlighting**

The flavonoid enzyme, chalcone isomerase, localizes to both the ER and the nucleus in Arabidopsis root cells. Fixed whole-mount seedlings were labeled with antibodies specific for CHI (red) and the ER-resident protein BiP (green), nuclei were counterstained with DAPI (blue), and samples were optically sectioned by confocal microscopy as described in Saslowsky *et al.*, 2005. Image by David E. Saslowsky.

**Figure:**
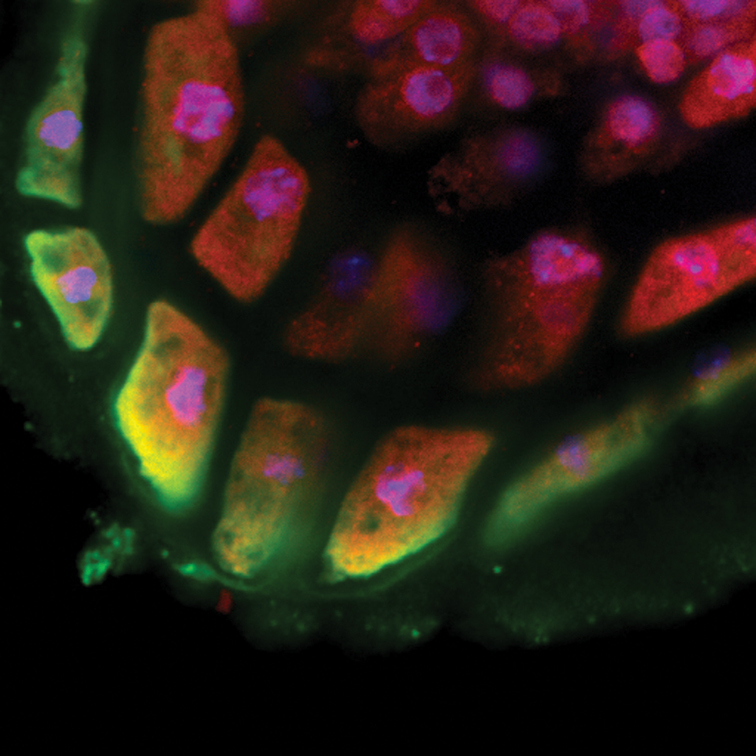

## A new dimension to moonlighting

One of the latest discoveries is described in the paper from Susanne Kohalmi’s group at Western University in Ontario (Bross *et al.*, 2017). This study adds a new spin to the idea of moonlighting, showing for the first time a connection between central metabolism and organelle division in plants, specifically components of the chloroplast division machinery. Arogenate dehydratases (ADTs) are found only in plants and some groups of microbes, where they play an important role in phenylalanine biosynthesis. The new study provides an in-depth analysis of the localization of the six Arabidopsis ADT isoforms, all of which are targeted, by virtue of a transit peptide, to the stroma and stromules of chloroplasts. This extends previous reports on the presence of these six proteins in chloroplasts, as also reported for four ADTs from *Pinus pinaster* (Rippert *et al.*, 2009; El-Azaz *et al.*, 2016). Bross *et al.* now show that two of the isoforms have additional localizations that point to non-catalytic functions. Confocal microscopy of constructs transiently expressed in tobacco and Arabidopsis (the latter an important technical innovation), together with insights from mutant analysis, suggest that ADT2 participates in the plastid division process as one of the as-yet-unidentified components of a plastid division ring. Similar analyses show that another isoform, ADT5, has an additional location in the nucleus, suggesting the intriguing possibility that it could be involved in retrograde signaling from the plastid to influence nuclear gene expression.

The isoform-specific differential roles of the plant ADTs raise the question of how and when such multifunctional entities might evolve within a group of closely related proteins. Comprehensive phylogenetic analyses previously classified the plant ADTs into four subgroups that are distinct from the groups encompassing microbial ADTs, including those in the Bacterioidetes/Chlorobi, which represent the closest bacterial homologs (Dornfeld *et al.*, 2014; El-Azaz *et al.*, 2016). This analysis further suggests that plant ADTs diversified following the split of plants and chlorophytes (green algae), with present-day ADTs having evolved from the subfamily I-type enzymes to which ADT2 belongs. This phylogeny further indicates that ADT2 is more closely related to ADT enzymes in other plants than its homologs in Arabidopsis, suggesting that its role in plastid division predates an early split in the green lineage. This phylogeny also indicates that ADT4 and ADT5 are closely related, offering the possibility for defining sequences involved in localization and/or nuclear function by comparative analysis.

## Why are enzymes so large? A new answer

It seems that alternative functions should be anticipated, if not expected, for most proteins and perhaps especially for enzymes of central metabolism. Three decades ago, in the title of a now-classic article in *Trends in Biochemical Sciences*, Paul Srere posed the question, ‘Why are enzymes so big?’ (Srere, 1984). Observing that the catalytic functions of enzymes required only a small portion of a typical protein’s overall dimensions, Srere proposed that the large size could reflect the need for sufficient surface area to direct localization and integration into metabolic complexes. Perhaps a corollary to this explanation is that enzyme size may also reflect the presence of, or relics of, alternative functions. In any case, a greater awareness that there may be more to enzymes, and other proteins, than we may first suspect will surely be crucial going forward, whether in studies of protein structure–function relationships or efforts to manipulate metabolism in plants and other organisms.

## References

[CIT0001] Bross CD Howes TR Abolhassani Rad S Kljakic O Kohalmi SE. 2017 . Subcellular localization of Arabidopsis arogenate dehydratases suggests novel and non-enzymatic roles. Journal of Experimental Botany 68 , 1425–1440. 28338876 10.1093/jxb/erx024 PMC5444438

[CIT0002] Commichau FM Stülke J. 2008 . Trigger enzymes: bifunctional proteins active in metabolism and in controlling gene expression. Molecular Microbiology 67 , 692 – 702 . 18086213 10.1111/j.1365-2958.2007.06071.x

[CIT0003] Dornfeld C Weisberg AJ K C R Dudareva N Jelesko JG Maeda HA. 2014 . Phylobiochemical characterization of class-ib aspartate/prephenate aminotransferases reveals evolution of the plant arogenate phenylalanine pathway. The Plant Cell 26 , 3101 – 3114 . 25070637 10.1105/tpc.114.127407 PMC4145135

[CIT0004] El-Azaz J de la Torre F Ávila C Cánovas FM. 2016 . Identification of a small protein domain present in all plant lineages that confers high prephenate dehydratase activity. The Plant Journal 87 , 215 – 229 . 27125254 10.1111/tpj.13195

[CIT0005] Frederikse PH Nandanoor A Kasinathan C. 2016 . “Moonlighting” GAPDH protein localizes with AMPA receptor GluA2 and L1 axonal cell adhesion molecule at fiber cell borders in the lens. Current Eye Research 41 , 41 – 49 . 25614994 10.3109/02713683.2014.997886

[CIT0006] He H Lee MC Zheng LL Zheng L Luo Y. 2013 . Integration of the metabolic/redox state, histone gene switching, DNA replication and S-phase progression by moonlighting metabolic enzymes. Bioscience Reports 33 , e00018 . 23134369 10.1042/BSR20120059 PMC3561917

[CIT0007] Henderson B Martin A. 2013 . Bacterial moonlighting proteins and bacterial virulence. Current Topics in Microbiology and Immunology 358 , 155 – 213 . 22143554 10.1007/82_2011_188

[CIT0008] Hildebrandt T Knuesting J Berndt C Morgan B Scheibe R. 2015 . Cytosolic thiol switches regulating basic cellular functions: GAPDH as an information hub? Biological Chemistry 396 , 523 – 537 . 25581756 10.1515/hsz-2014-0295

[CIT0009] Jeffery CJ. 2014 . An introduction to protein moonlighting. Biochemical Society Transactions 42 , 1679 – 1683 . 25399589 10.1042/BST20140226

[CIT0010] Keating ST El-Osta A. 2015 . Epigenetics and metabolism. Circulation Research 116 , 715 – 736 . 25677519 10.1161/CIRCRESAHA.116.303936

[CIT0011] Latrasse D Jégu T Meng PH . 2013 . Dual function of MIPS1 as a metabolic enzyme and transcriptional regulator. Nucleic Acids Research 41 , 2907 – 2917 . 23341037 10.1093/nar/gks1458 PMC3597657

[CIT0012] Li L Xu XH Chen C Shen ZG. 2016 . Genome-wide characterization and expression analysis of the germin-like protein family in rice and Arabidopsis. International Journal of Molecular Sciences 17 , 1622 . 10.3390/ijms17101622 PMC5085655 27669230

[CIT0013] Marondedze C Thomas L Serrano NL Lilley KS Gehring C. 2016 . The RNA-binding protein repertoire of *Arabidopsis thaliana*. Scientific Reports 6 , 29766 . 27405932 10.1038/srep29766 PMC4942612

[CIT0014] Rippert P Puyaubert J Grisollet D Derrier L Matringe M. 2009 . Tyrosine and phenylalanine are synthesized within the plastids in Arabidopsis. Plant Physiology 149 , 1251 – 1260 . 19136569 10.1104/pp.108.130070 PMC2649395

[CIT0015] Rolland F Baena-Gonzalez E Sheen J. 2006 . Sugar sensing and signaling in plants: conserved and novel mechanisms. Annual Review of Plant Biology 57 , 675 – 709 . 10.1146/annurev.arplant.57.032905.105441 16669778

[CIT0016] Saslowsky DE Warek U Winkel BS. 2005 . Nuclear localization of flavonoid enzymes in *Arabidopsis*. The Journal of Biological Chemistry 280 , 23735 – 23740 . 15817473 10.1074/jbc.M413506200

[CIT0017] Sirover MA. 2014 . Structural analysis of glyceraldehyde-3-phosphate dehydrogenase functional diversity. The International Journal of Biochemistry & Cell Biology 57 , 20 – 26 . 25286305 10.1016/j.biocel.2014.09.026 PMC4268148

[CIT0018] Srere PA. 1984 . Why are enzymes so big? Trends in Biochemical Sciences 9 , 387 – 390 .

[CIT0019] Su XB Pillus L. 2016 . Functions for diverse metabolic activities in heterochromatin. Proceedings of the National Academy of Sciences, USA 113 , E1526 – E1535 . 10.1073/pnas.1518707113 PMC4801238 26936955

[CIT0020] Toleman C Paterson AJ Whisenhunt TR Kudlow JE. 2004 . Characterization of the histone acetyltransferase (HAT) domain of a bifunctional protein with activable *O*-GlcNAcase and HAT activities. The Journal of Biological Chemistry 279 , 53665 – 53673 . 15485860 10.1074/jbc.M410406200

[CIT0021] Torres-Machorro AL Aris JP Pillus L. 2015 . A moonlighting metabolic protein influences repair at DNA double-stranded breaks. Nucleic Acids Research 43 , 1646 – 1658 . 25628362 10.1093/nar/gku1405 PMC4330366

[CIT0022] Vega M Riera A Fernández-Cid A Herrero P Moreno F. 2016 . Hexokinase 2 is an intracellular glucose sensor of yeast cells that maintains the structure and activity of Mig1 protein repressor complex. The Journal of Biological Chemistry 291 , 7267 – 7285 . 26865637 10.1074/jbc.M115.711408 PMC4817161

[CIT0023] Warner JR McIntosh KB. 2009 . How common are extraribosomal functions of ribosomal proteins? Molecular Cell 34 , 3 – 11 . 19362532 10.1016/j.molcel.2009.03.006 PMC2679180

[CIT0024] Zaffagnini M Fermani S Costa A Lemaire SD Trost P. 2013 . Plant cytoplasmic GAPDH: redox post-translational modifications and moonlighting properties. Frontiers in Plant Science 4 , 450 . 24282406 10.3389/fpls.2013.00450 PMC3824636

